# County-level land use carbon budget dynamics and heterogeneous driving mechanisms in Chengdu plain, China: integrating FLUS and GTWR model

**DOI:** 10.1186/s13021-026-00467-9

**Published:** 2026-06-11

**Authors:** Qiang Huang, Guangjie Wang, Lei Hu, Jiahui Xie

**Affiliations:** 1 The Institute of Geography and Resources Science, Sichuan Normal University, Chengdu, 610068 China; 2 Key Lab of Land Resources Evaluation and Monitoring in Southwest, Ministry of Education, Sichuan Normal University, Chengdu, 610068 China

**Keywords:** Land use carbon budget, FLUS model, GTWR model, Spatiotemporal heterogeneity, Chengdu plain

## Abstract

**Background:**

Against the backdrop of global warming, land use change critically shapes regional carbon budgets by altering both emission sources and carbon sinks. Existing studies predominantly focus on macro-scales, overlooking county-level heterogeneity and future trajectories, thus limiting precise carbon balance management. The Chengdu Plain, as a key economic agglomeration and upper Yangtze ecological barrier, faces severe carbon imbalance challenges due to rapid urbanization. This study contributes three novel insights: (i) integrating county-scale analysis with future land use simulation to project carbon budget dynamics; (ii) applying GTWR to reveal spatiotemporally heterogeneous driving mechanisms that macro-scale studies mask; and (iii) developing a county typology-based carbon management framework.

**Results:**

(1) From 2000 to 2030, construction land expanded significantly while cropland, forest, and grassland continuously contracted; (2) The net carbon budget (net emissions) followed a trajectory of initial increase and subsequent decline, peaking at 6673.122 × 10⁴ t in 2020 before declining to a projected 6409.177 × 10⁴ t by 2030. Construction land was the dominant carbon source, while forests served as the primary carbon sink, together accounting for over 95% of the gross budget; (3) County-level net carbon budgets exhibited strong positive spatial autocorrelation, dominated by High-High and Low-Low agglomeration patterns; (4) GTWR results indicated that consumption level (mean coefficient: 0.466), urbanization level (0.392), population size (0.388), and industrial structure (0.196) positively drove net carbon emissions, while investment level (-0.048) exhibited an inhibiting effect not captured in average macro-scale models. Spatiotemporal heterogeneity was notable.

**Conclusion:**

Achieving a managed regional carbon balance requires differentiated county-level strategies: For urban core counties, low-carbon community transformation and optimized urban layout; for industrial counties, carbon intensity thresholds and green industrial land policies; for underdeveloped southwest counties, green consumption promotion; and cross-regional carbon compensation mechanisms to strengthen sink protection. This county-scale, mechanism-based framework provides a scientific toolkit for precise carbon balance management in ecologically and economically vital regions.

## Introduction

In recent years, the increase in carbon emissions caused by global economic development has made the issue of global climate warming increasingly prominent, and climate change has received growing attention [1]. This not only poses a serious threat to the high-quality development of the ecological environment but also has a negative impact on the sustainable development of human society [2]. As the world’s largest developing country, a permanent member of the UN Security Council, and the world’s second-largest economy, China actively fulfills its responsibilities as a major power, responds to the international community’s call for carbon emission reduction, and commits to achieving carbon peak by 2030 and carbon neutrality by 2060, known as the “dual carbon” goals [3, 4]. The United Nations Intergovernmental Panel on Climate Change (IPCC) pointed out that approximately one-third of global anthropogenic carbon emissions between 1950 and 1998 were attributed to land development and utilization [5, 6]. Critically, land use change not only directly increases anthropogenic carbon emissions but also alters the regional carbon budget by weakening ecosystem carbon sink capacity—through deforestation, wetland conversion, and cropland expansion—thereby creating a dual pressure on climate mitigation efforts. Against this backdrop, in-depth research on land use carbon emissions is of great significance for promoting regional carbon emission reduction and sustainable development.

Currently, scholars both domestically and internationally have conducted extensive research on carbon emissions from land use change. The existing research on carbon emissions from land use shows significant characteristics of scale bias and content focus: in terms of spatial scale, it mainly focuses on macro-scales such as countries, provinces, and urban agglomerations, while research at the micro-scale is relatively scarce; in terms of research content, it mostly concentrates on the description of spatiotemporal patterns and the identification of driving factors, but there is insufficient exploration of the internal heterogeneity within regions and the interactions between units. Domestically, Yang et al. re-estimated the carbon emissions caused by land use and land cover change (LUCC) in China from 1700 to 1980 by updating the accounting model, and analyzed the total amount, main contributing factors, and spatial distribution [7]. Many scholars (e.g., Peng et al. [8]) have explored the spatiotemporal differentiation and influencing factors of carbon emissions at the provincial level using carbon emission measurement models and various econometric approaches (such as STIRPAT and GWR models), revealing carbon emission patterns at the municipal scale within provinces [8–11]. Scholars (e.g., Deng et al. [12]) have examined the spatiotemporal evolution, patterns, and spatial distribution of land use carbon emissions in regions such as the Wuhan Metropolitan Circle and the Beijing-Tianjin-Hebei Urban Agglomeration, and analyzed their driving factors and environmental parameters at the municipal scale within urban agglomerations [12–15]. Internationally, scholars such as Dutra et al. assessed the challenges faced by carbon emissions from land use in Brazil at the national level and proposed solutions for protecting and restoring Brazilian biomes to achieve carbon emission reduction [16]; Carpio et al. analyzed the relationship between urban expansion and carbon emissions in the Monterrey Metropolitan Area of Mexico from 1990 to 2019, considering the impact of multiple variables such as population, urban expansion, and GDP on carbon emissions [17]; Winkler et al. studied at the international level and found that carbon sinks in Eastern Europe are declining, and based on the random forest model, demonstrated that changes in land use and management are the main driving factors for the decline of carbon sinks in Eastern Europe [18]. The above-mentioned studies mainly focus on the macro-scale. Although they can reflect provincial differences, intra-provincial municipal differences, and inter-city interactions in carbon emissions, they overlook the detailed heterogeneity of counties under prefecture-level cities. For example, the carbon emission driving mechanisms in economically underdeveloped counties and industrial counties within the same municipality may be completely different. Macro-analysis is prone to cause the “averaging bias” and is difficult to support precise carbon emission reduction policies at the county level. As the basic unit of China’s territorial spatial planning, the differentiated understanding of the carbon emission characteristics of counties plays a crucial role in achieving the “dual-carbon” goal. On the other hand, most of the research content remains at the “description of spatiotemporal patterns” and “identification of driving factors” at the provincial and municipal levels for known years, failing to reflect the differences in spatiotemporal distribution and driving factors at the county level and the future change trends of land use carbon emissions. As the basic unit of regional development, there must be significant internal differences in the land use carbon emissions of counties. Conducting research at the county level can more meticulously analyze the heterogeneous characteristics and internal relationships of regions in terms of land use carbon emissions, which is crucial for formulating precise and effective carbon emission reduction strategies and land use plans.

The Chengdu Plain, located in the southwest of China, is an important economic, cultural, demographic, and industrial agglomeration area in the southwest region, as well as a crucial ecological barrier in the upper reaches of the Yangtze River, occupying a pivotal position in regional development and ecological protection [19]. In recent years, the acceleration of urbanization and industrialization in the Chengdu Plain has led to rapid economic development. At the same time, it has also caused profound changes in land use patterns, with continuous expansion of construction land and reduction in the area of arable land and forest land. These changes have triggered multiple carbon-related environmental issues: (1) intensified regional greenhouse effect and frequent extreme weather [19, 20]; (2) encroachment on carbon sinks such as forest land and wetlands, weakening ecosystem carbon sequestration capacity [8, 21]; and (3) increased energy consumption and pollutant emissions that indirectly threaten soil, water, and air quality [22].

Given the Chengdu Plain’s dual role as an economic engine and ecological barrier in the upper Yangtze River, understanding its county-level carbon budget dynamics is critical for both regional sustainability and downstream climate resilience. This study offers three distinct contributions that extend beyond existing macro-scale research: (i) Spatial scale refinement with future pathway projection. By coupling county-level carbon accounting with the FLUS model, we capture a projected carbon peak (circa 2020) and subsequent decline that provincial studies often miss, thereby identifying a critical management window. (ii) Mechanism heterogeneity uncovered. Applying GTWR to panel data across 43 counties, we reveal that key drivers—consumption level, urbanization level, population size, industrial structure, and notably investment level—exert spatially and temporally heterogeneous effects, including an emission-inhibiting role of investment that contradicts previous average-effect findings. (iii) From analysis to carbon balance management. We translate the identified emission-sink dynamics and driving heterogeneity into a county typology (urban-core, industrial-dominant, agro-ecological) linked with differentiated policy instruments. This framework directly addresses the pressing need for spatially explicit carbon management strategies in rapidly urbanizing yet ecologically critical regions, offering a transferable paradigm for other major basin areas.

## Methodology

### Study area

**Fig. 1:**
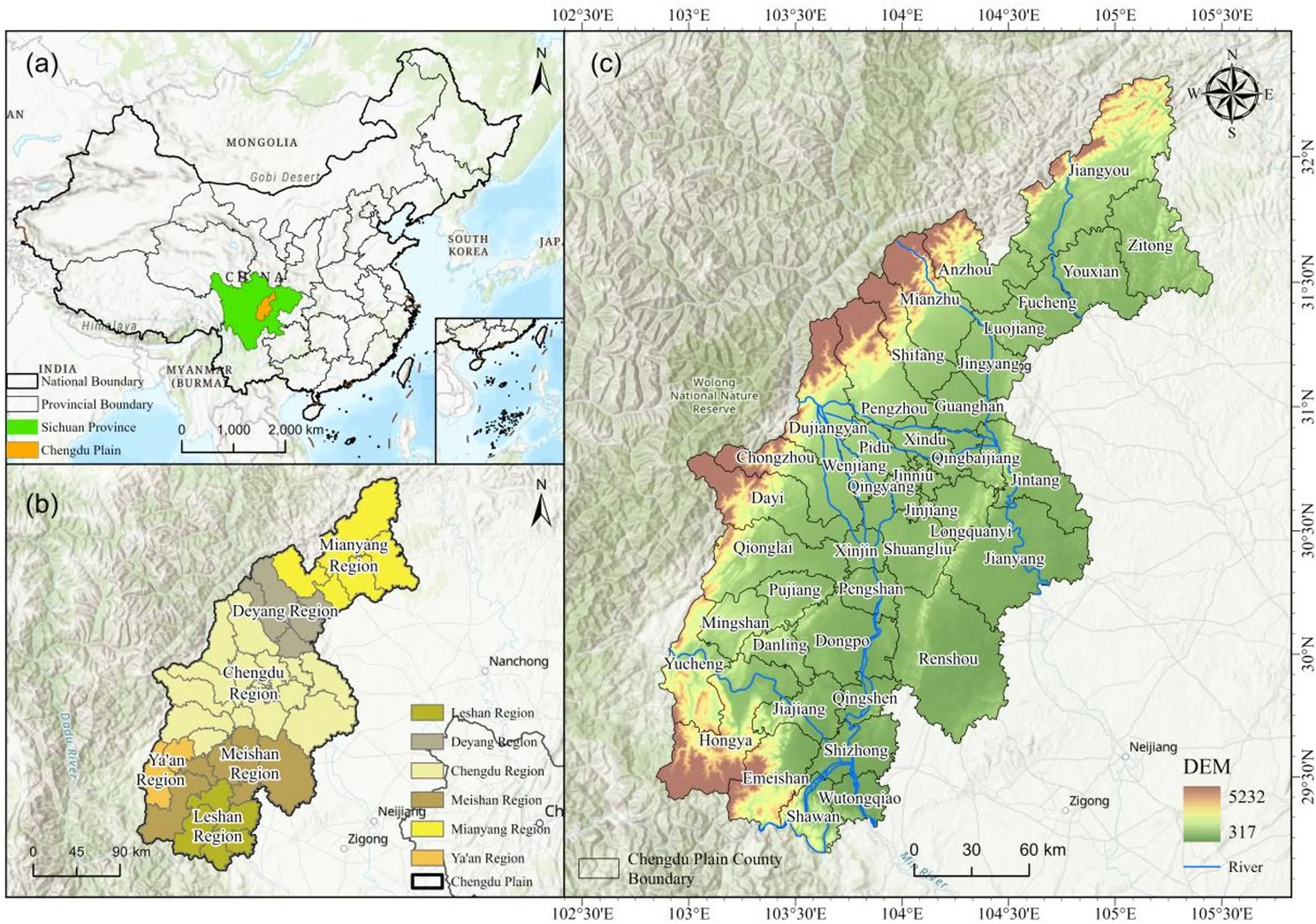
Location of the Chengdu Plain. (**a**) Geographical location of the Chengdu Plain in China; (**b**) Administrative division of the study area, marking the 43 counties/districts under 6 prefecture-level cities; (**c**) Topography and river network distribution of the study area

The Chengdu Plain is located in the central part of Sichuan Province, China, and lies in the core area of the Sichuan Basin (Fig. 1(a)). Geographically, it ranges from 102°50′ to 105°27′ east longitude and 29°11′ to 32°19′ north latitude, covering 43 districts and counties in 6 prefecture-level cities such as Chengdu, Mianyang, and Deyang (Fig. 1(b)), with a total area of about 37,600 square kilometers. The terrain within the area is flat, sloping from the northwest to the southeast (Fig. 1(c)). The region has a subtropical monsoon climate, with an average annual temperature of about 18 °C and an average annual precipitation of over 1000 millimeters [19]. The water and heat conditions support a land cover mainly composed of forest land (mostly artificial forests) and cultivated land. The flat terrain formed by the alluvial deposits of the Minjiang River and the Tuojiang River, along with the purple soil, makes the cultivated land highly sensitive to carbon emissions from agricultural activities. Meanwhile, the wetland ecosystem nurtured by the dense river network serves as a supplementary part of the carbon sink. As an important ecological barrier in the upper reaches of the Yangtze River, its unique natural characteristics provide a special background for the carbon cycle process. The balance between carbon sources and sinks in this area not only directly affects the stability of the regional ecosystem but also plays an irreplaceable strategic supporting role in achieving the “dual-carbon” goals of the Yangtze River Basin and even the whole country.

Economically, the plain serves as a vital hub in the southwest, showcasing a diverse and dynamic industrial landscape. Chengdu, its core city, has seen rapid growth in high-tech sectors like electronic information, biomedicine, and high-end equipment manufacturing. Many well-known enterprises have set up operations here, forming complete industrial chains that drive employment and generate substantial economic value. Beyond Chengdu, Mianyang’s science and technology industry, Deyang’s equipment manufacturing, and Meishan and Leshan’s specialized chemical and food/beverage sectors each demonstrate strong competitiveness in their fields, propelling the region’s economic expansion. Commercially, the area thrives with a robust trade environment. Modern services such as transportation, finance, and logistics are continuously improving and upgrading, which has elevated the Chengdu Plain’s standing within China’s national economic framework.

It should be noted that in delineating the study area for this research, some mountainous regions were included. This was primarily done to maintain the integrity of administrative divisions and facilitate data statistics and analysis.

### Data sources and research methods

#### Data sources and preprocessing

This study utilizes a dataset primarily consisting of land use data for the Chengdu Plain in 2000, 2010, and 2020, alongside corresponding natural factor and socio-economic datasets (Table 1 for details). Researchers obtained the land use data from US Landsat satellite remote sensing imagery—including Landsat MSS, TM/ETM, and Landsat8—through human-computer interactive visual interpretation. These data were sourced from the Resource and Environmental Science Data Center of the Chinese Academy of Sciences (http://www.resdc.cn) [23]. Using ArcMap 10.8 software, analysts reclassified the land use data into six major categories: cropland, forestland, grassland, water bodies, construction land, and unused land. The natural factor dataset includes a Digital Elevation Model (DEM), Normalized Difference Vegetation Index (NDVI), river distribution, temperature, and precipitation. The socio-economic dataset incorporates indicators such as population size, Gross Domestic Product (GDP), road networks, railway lines, number of railway stations, and energy consumption.

To derive slope information, the study used the slope analysis module within ArcMap’s surface analysis functions, applying DEM data as the foundation. River distribution data were generated through the hydrological analysis module in ArcMap, also relying on DEM inputs. Euclidean distance analyses were performed on river, road, railway, and railway station datasets, producing separate distance layers for rivers, main roads, secondary roads, highways, railways, and railway stations. Construction land data, extracted via land use reclassification, underwent similar Euclidean distance analysis to create a construction land distance dataset. Given the study’s 100-meter resolution scale, all datasets were resampled in ArcMap prior to analysis to ensure uniform 100-meter resolution across the board.

**Table 1:** Data Sources

Data Type	Name	Year	Spatial Resolution	Unit	Data Source
Land Use Data	Land Use	2000/2010/2020	30 m		Resource and Environmental Science Data Center, Chinese Academy of Sciences (http://www.resdc.cn)
Administrative Division Data	Administrative Division	2024			National Geographic Information Public Service Platform (Tianditu) (https://www.tianditu.gov.cn/)
Natural Factor Data	DEM		30 m	m	NASA SRTM Digital Elevation 30 m
	NDVI	2010/2020	30 m		Land Use and Global Change Remote Sensing Team, Institute of Geographic Sciences and Natural Resources Research, Chinese Academy of Sciences, obtained via Google Earth Engine cloud computing platform [24]
	River		30 m		Hydrological analysis of DEM in ArcMap
	Slope		30 m	°	Slope analysis of DEM in ArcMap
	Annual Mean Temperature	2010/2020	1000 m	℃	National Tibetan Plateau Data Center (https://data.tpdc.ac.cn/home)
	Annual Precipitation	2010/2020	1000 m	mm	
Socio-economic Data	Population	2010/2020	1000 m	person	Population dataset from Landscan platform (https://landscan.ornl.gov)
	GDP	2010/2020	1000 m	million	Resource and Environmental Science Data Center, Chinese Academy of Sciences (http://www.resdc.cn)
	Road	2010/2020			Open street map (https://www.openstreetmap.org/)
	Railway	2010/2020			
	Railway Station	2010/2020			
	Energy Consumption	2000/2010/2020		t	*Sichuan Statistical Yearbook*

#### Research methods

##### Land use carbon emissions estimation

This study focuses on estimating carbon emissions and carbon sequestration from cropland, forestland, grassland, water areas, unused land, and construction land. In terms of cropland, crops absorb CO₂ from the atmosphere through photosynthesis, but their respiration releases the absorbed CO₂ back into the atmosphere in the short term, making their function as a carbon sink less significant [25]. Therefore, this study regards construction land and cropland as major carbon sources, while forestland, grassland, water areas, and unused land are considered major carbon sinks (carbon sequestration). Additionally, carbon emissions from land use include direct land use carbon emissions (i.e., carbon emissions caused by changes in land use structure) and indirect land use carbon emissions (i.e., carbon emissions generated during land use activities) [26]. Based on this, this study proposes the formula for estimating carbon emissions from land use as follows:

1 $$\:\mathrm{C=}{\mathrm{C}}_{\mathrm{x}}\mathrm{+}{\mathrm{C}}_{\mathrm{y}}$$

In the formula, *C* represents the total carbon emissions, *C*_*x*_ represents the indirect land use carbon emissions. Since construction land hosts the majority of human production activities, the indirect land use carbon emissions in this study refer to carbon emissions from construction land. *C*_*y*_ represents the sum of carbon emissions (carbon sequestration) from five land types: cropland, forestland, grassland, water areas, and unused land, which can be estimated using the IPCC carbon emission coefficient method. The calculation formula can be expressed as follows:

2 $$\:{\mathrm{C}}_{\mathrm{y}}\mathrm{=}\sum\:{\mathrm{S}}_{\mathrm{i}}\times{\delta}_{\mathrm{i}}$$

In the formula, *C*_*y*_ represents the direct carbon emissions from land use; *S*_*i*_ represents the area of the *i*-th land use type; *δ*_*i*_ represents the carbon emission (carbon sequestration) coefficient of the *i*-th land use type. With reference to existing literature research results [27], the carbon emission (carbon sequestration) coefficients (t/hm^2^) for the five land types in this study are shown in Table 2. A positive coefficient indicates that the land type is mainly a carbon source, while a negative coefficient indicates a carbon sink.

**Table 2:** Land Use Carbon Emission Coefficient (t/hm^2^)

Land Use Type	Cropland	Forestland	Grassland	Water Areas	Unused Land
Coefficient	0.469	−0.605	−0.021	−0.253	−0.003

Generally, carbon emissions from construction land are indirectly estimated by calculating the carbon emissions generated from energy consumption during the utilization of construction land. The calculation formula can be expressed as:

In the formula, *C*_*x*_ *represents* the carbon emissions from construction land; *n* represents the total number of energy types; *Q*_*i*_ represents the consumption of the *i*-th energy type; *N*_*i*_ represents the carbon emission coefficient of the *i*-th energy type; *M*_*i*_ represents the standard coal equivalent coefficient of the *i*-th energy type. Based on the actual energy usage in Sichuan Province and the Chengdu Plain, the energy types selected in this study include natural gas, diesel oil, kerosene, gasoline, fuel oil, crude oil, coke, raw coal, cleaned coal, other washed coal, and electricity (Table 3). With reference to existing literature [28], the values of the standard coal equivalent coefficients and carbon emission coefficients for different energy types are shown in Table 3.

**Table 3:** Standard Coal Coefficient and Carbon Emission Coefficient

Energy Type	Standard Coal Coefficient (kgce/kg)	Carbon Emission Coefficient (t C/t)
Natural Gas	1.214	0.448
Diesel Oil	1.457	0.592
Kerosene	1.471	0.571
Gasoline	1.471	0.554
Fuel Oil	1.429	0.619
Crude Oil	1.429	0.586
Coke	0.971	0.855
Raw Coal	0.714	0.756
Cleaned Coal	0.900	0.756
Other Washed Coal	0.286	0.756
Electricity	0.123	0.733

The unit of standard coal coefficient for natural gas is kgce/m^3^, and for electricity is kgce/kWh

Due to the lack of energy consumption statistics for 43 districts and counties in the Chengdu Plain region, this study employs the energy consumption per unit of GDP method [28] to estimate carbon emissions from county-level construction land. Specifically, it allocates energy consumption to county-level scales based on each district’s GDP proportion, using the energy consumption per unit of GDP of Sichuan Province or prefecture-level cities as the baseline [28–30]. The calculation formula is expressed as:

4 $$\mathrm{D}_{\mathrm{i}}=\frac{\mathrm{E}_{\mathrm{i}}}{\mathrm{GDP}_{\mathrm{i}}}$$

5 $$\:{\mathrm{C}}_{\mathrm{i}}\mathrm{=}{\mathrm{C}}_{\mathrm{x}}\times\left({\mathrm{E}}_{\mathrm{i}} \div \sum\:{\mathrm{E}}_{\mathrm{i}}\right)\mathrm{=}{\mathrm{C}}_{\mathrm{x}}\times\left[\left({\mathrm{GDP}}_{\mathrm{i}}\times{\mathrm{D}}_{\mathrm{i}}\right)\div\left(\sum\:\left({\mathrm{GDP}}_{\mathrm{i}}\times{\mathrm{D}}_{\mathrm{i}}\right)\right)\right]$$

In the formula, *C*_*i*_ represents the carbon emissions from construction land in the *i*-th district or county, with the unit of t; *E*_*i*_ represents the total energy consumption in the *i*-th district or county, with the unit of t; *GDPi* represents the gross domestic product of the *i*-th district or county in that year; and *Di* represents the energy consumption per unit of GDP in the *i*-th district or county.

To predict the carbon emissions from land use in 2030, it is necessary to clarify the GDP data and energy consumption data for that year. This paper uses linear regression analysis based on time series to construct a prediction model for GDP and energy consumption in 2030. The calculation formula is as follows:

6 $$\:y=\:{\beta\:}_{0}+\:{\beta\:}_{1}{X}_{1}+{\beta\:}_{2}{X}_{2}+\cdots\:+{\beta\:}_{P}{X}_{P}+\epsilon\:$$

In the formula: *y* is the dependent variable; *X*_1_, *X*_2_ … *X*_P_ are the independent variables; *β*_0_ is the constant term; *β*_1_, *β*_2_ … *β*_*p*_ are the regression parameters; and $\:\epsilon\:$ is the error term. When predicting the GDP data for 2030 in this paper, the time points (from 2000 to 2020, a total of 20 time points) are used as the independent variables, and the GDP is used as the dependent variable. When predicting the energy consumption data, the time points (from 2008 to 2022, a total of 15 time points) are used as the independent variables, and the energy consumption of each type is used as the dependent variable.

##### Future land use simulation (FLUS) model

The Future Land Use Simulation Model (FLUS Model) is a prediction tool that adopts neural network algorithms. By calculating the suitability probability of land use driven by multiple factors such as nature and humanity, the model effectively simulates and predicts the evolution trend of future urban land use [31]. The operation process of the FLUS Model includes: first, using the Markov chain algorithm to determine the simulated target quantity at the end of the forecast period [32]; second, using the BP-ANN algorithm to evaluate the suitability probability of different land use types under multiple driving factors [33]; finally, under the constraint of the adaptive inertia mechanism, the CA model is used to complete the simulation of the future land use spatial layout [34].

When conducting the simulation study on the suitability probability of land use driving factors, this research selected 12 key factors, including Digital Elevation Model (DEM), slope, Normalized Difference Vegetation Index (NDVI, maximum value), annual average temperature, annual precipitation, distance to the nearest river, distance to the nearest expressway, distance to the nearest main road, distance to the nearest secondary road, distance to the nearest railway, distance to the nearest high-speed rail station, and distance to the nearest built-up area. All selected driving factors underwent unified resampling processing to achieve a spatial resolution of 100 m and were normalized. Using the data from 2010 as training samples, this study simulated the land use suitability probability and distribution pattern in the Chengdu Plain for 2020, and compared them with the actual land use data of the Chengdu Plain in 2020. Under the condition of 5% random sampling, the model’s Kappa coefficient and overall accuracy reached 0.780 and 0.870, respectively; under the condition of 10% random sampling, the Kappa coefficient and overall accuracy were 0.777 and 0.868, respectively; under the condition of 10‰ uniform sampling, the Kappa coefficient and overall accuracy were 0.760 and 0.800, respectively; and under the condition of 20‰ uniform sampling, the Kappa coefficient and overall accuracy were 0.771 and 0.809, respectively. In all test scenarios, the model passed the accuracy verification, demonstrating high prediction accuracy and indicating that the model has the potential to predict the land use distribution pattern in the Chengdu Plain for 2030.

During the model prediction process, the conversion of different land use types is not only affected by the weight of neighborhood factors but also closely related to the land use transition matrix. Referring to relevant studies and considering the actual situation of the region, after multiple rounds of debugging, the neighborhood weights of cultivated land, forest land, grassland, water area, construction land, and unused land in this study were set to 0.8, 0.8, 0.2, 0.4, 1, and 0.2 respectively [35–37]. The rule matrix of conversion costs among various land - use types is detailed in Table 4 [20, 35, 38].

**Table 4:** Matrix of land use conversion cost rules

Cropland	Cropland	Forestland	Grassland	Water Areas	Construction Land	Unused Land
	1	0	0	1	1	0
Forestland	1	1	1	0	1	1
Grassland	1	1	1	0	1	1
Water Areas	1	0	0	1	1	0
Construction Land	0	0	0	0	1	0
Unused Land	1	1	1	1	1	1

0 indicates that conversion is not allowed, and 1 indicates that conversion is allowed; in the matrix, rows represent conversion out, and columns represent conversion in

##### Spatial correlation indicator (Moran’s *I* Index)

The analysis of spatial autocorrelation aims to explore the interaction between spatial objects, encompassing two dimensions: global spatial autocorrelation and local spatial autocorrelation [39, 40]. As a commonly used tool for studying spatial correlation issues, Moran’s *I* index is mainly applied to the quantitative assessment of the degree of agglomeration or dispersion of spatial elements [41]. Compared to other analytical methods, Moran’s *I* index demonstrates more significant advantages in terms of flexibility in spatial weights, explanatory power, and statistical significance testing [42], and it is divided into global Moran’s *I* index and local Moran’s *I* index. In this study, the global Moran’s *I* index is mainly used to reveal the overall spatial distribution characteristics of land use carbon emissions. Its value ranges between [−1,1], with positive values indicating positive correlations between elements and negative values indicating negative correlations. The calculation formula is expressed as follows:

7 $$\:\mathrm{I=}\frac{\mathrm{n}\sum\:_{\mathrm{i=1}}^{\mathrm{n}}\sum\:_{\mathrm{j=1}}^{\mathrm{n}}{\omega}_{\mathrm{ij}}\mathrm{(}{\mathrm{x}}_{\mathrm{i}}\mathrm{-}\stackrel{\mathrm{-}}{\mathrm{x}}\mathrm{)(}{\mathrm{x}}_{\mathrm{j}}\mathrm{-}\stackrel{\mathrm{-}}{\mathrm{x}}\mathrm{)}}{\sum\:_{\mathrm{i=1}}^{\mathrm{n}}\sum\:_{\mathrm{j=1}}^{\mathrm{n}}{\omega}_{\mathrm{ij}}\sum\:_{\mathrm{i=1}}^{\mathrm{n}}{\mathrm{(}{\mathrm{x}}_{\mathrm{i}}\mathrm{-}\stackrel{\mathrm{-}}{\mathrm{x}}\mathrm{)}}^{\mathrm{2}}}$$

In the formula, *I* represents the global Moran’s *I* index value; *n* represents the number of studied counties; *x*_*i*_ and *x*_*j*_ represent the carbon emissions of two adjacent counties in space, respectively; *ω*_*ij*_ represents the spatial adjacency weight between county *i* and *j*; and $\:\stackrel{-}{x}$ represents the average carbon emissions of all county units in the study area.

The local Moran’s *I* index, on the other hand, is used to reflect the spatial interrelationship between the carbon emissions of each county and those of its surrounding counties, thereby revealing the agglomeration or dispersion state of elements at the local level. Its calculation formula can be expressed as:

8 $$\:{\mathrm{I}}_{\mathrm{i}}\mathrm{=}\frac{\mathrm{n(}{\mathrm{x}}_{\mathrm{i}}\mathrm{-}\stackrel{\mathrm{-}}{\mathrm{x}}\mathrm{)}\sum\:_{\mathrm{j=1}}^{\mathrm{n}}{\omega}_{\mathrm{ij}}\mathrm{(}{\mathrm{x}}_{\mathrm{j}}\mathrm{-}\stackrel{\mathrm{-}}{\mathrm{x}}\mathrm{)}}{\sum\:_{\mathrm{i=1}}^{\mathrm{n}}{\mathrm{(}{\mathrm{x}}_{\mathrm{i}}\mathrm{-}\stackrel{\mathrm{-}}{\mathrm{x}}\mathrm{)}}^{\mathrm{2}}}$$

In the formula, *I*_*i*_ represents the Moran’s *I* index of unit *i*; *n* represents the number of studied counties; *x*_*i*_ and *x*_*j*_ represent the carbon emissions of two adjacent counties in space, respectively; *ω*_*ij*_ represents the spatial adjacency weight between county *i* and *j*; and $\:\stackrel{-}{x}$ represents the average carbon emissions of all county units in the study area.

##### Geographically and temporally weighted regression model and data processing

1. Geographically and temporally weighted regression model

In the field of spatial heterogeneity research, the Geographically Weighted Regression model (GWR) is a classic and widely applied method. However, GWR has certain limitations in practical applications. This model mainly relies on cross-sectional data. Although the abnormal fluctuations of indicators can be alleviated by using the average values of multi-year data, it is still difficult in essence to depict the temporal dynamic characteristics of variable relationships. Meanwhile, the sample size of cross-sectional data is usually limited. When there are many explanatory variables, if it is necessary to incorporate the heterogeneity of their spatial spillover effects and construct a model containing spatial lag terms, estimation difficulties may occur due to an excessive number of parameters [43–45]. The proposal of the Geographically and Temporally Weighted Regression model (GTWR) provides a new research path for solving the above problems. The GTWR model introduces the time dimension on the basis of GWR, enabling the model to capture both temporal and spatial information simultaneously. From a theoretical perspective, GTWR breaks through the limitation of GWR that only considers spatial effects and provides a new methodology for testing spatial heterogeneity; from an empirical perspective, GTWR overcomes the limitation of GWR when the sample size is limited and improves the efficiency of estimation. The mathematical expression of the Geographically and Temporally Weighted Regression model is as follows:

9 $$\:{\mathrm{y}}_{\mathrm{i}}\mathrm{=}{\beta}_{\mathrm{0}}\left({\mathrm{u}}_{\mathrm{i}}{\mathrm{,v}}_{\mathrm{i}}{\mathrm{,t}}_{\mathrm{i}}\right)\mathrm{+}\sum\:_{\mathrm{k=1}}^{\mathrm{p}}{\beta}_{\mathrm{k}}\left({\mathrm{u}}_{\mathrm{i}}{\mathrm{,v}}_{\mathrm{i}}{\mathrm{,t}}_{\mathrm{i}}\right){\mathrm{x}}_{\mathrm{ik}}\mathrm{+}{\epsilon}_{\mathrm{i}}$$

In the formula, *y*_*i*_ is the dependent variable of the *i*-th sample point; *u*_*i*_ and *v*_*i*_ represent the longitude and latitude coordinates of the *i*-th sample point, respectively; (*u*_*i*_, *v*_*i*_, *t*_*i*_) represents the spatial-temporal coordinates of the *i*-th sample point; *β*_*0*_(*u*_*i*_, *v*_*i*_, *t*_*i*_) represents the regression constant of the *i*-th sample point, which is the constant term in the geographically and temporally weighted regression; *β*_*k*_(*u*_*i*_, *v*_*i*_, *t*_*i*_) represents the *k*-th regression parameter of the *i*-th sample point; *x*_*ik*_ represents the value of the independent variable *x*_*k*_ at point *i*, which is the value of each quantitative indicator in the indicator system of the geographically and temporally weighted regression model; and *ε*_*i*_ represents the residual term of the model [46].

(2) Selection and preprocessing of influencing factors

Considering economic, population, and technological dimensions alongside the specific context of the Chengdu Plain, this research identifies eight key indicators as influencing factors: the economic development level (measured by Gross Domestic Product (GDP)), the industrial structure (represented by “the proportion of the secondary-industry output value in GDP”), the urbanization level (measured by “the proportion of the urban population in the total population”), the population size (referring to “the total permanent resident population at the end of the year in the county”), the population density (calculated by “the number of permanent residents at the end of the year/the administrative area of the county”), the land use structure (represented by “the proportion of the construction land area in the total area of the county”), the consumption level (represented by “the per-capita total retail sales of social consumer goods”), and the investment level (represented by “the per-capita investment in fixed assets”) (Table 5) [47, 48]. All relevant data originate from statistical yearbooks published by prefectural-level city statistical departments. To address potential multicollinearity among these factors, analysts used SPSS software to test each influencing variable. Initial results indicated that variance inflation factors (VIFs) for economic development level, population density, and the proportion of construction land exceeded 7.5, with tolerance values below 0.1—signaling severe multicollinearity among these three. To mitigate this impact, researchers excluded these factors. A subsequent collinearity test on the remaining five variables showed VIFs below 7.5 and tolerance values above 0.1, confirming no significant collinearity issues. After applying Z-score standardization to the remaining influencing factors, the study constructed a geographically and temporally weighted regression model. This model uses the standardized independent variables and sets standardized carbon emissions as the dependent variable.

**Table 5:** Variables Influencing Land Use Carbon Emissions

Influencing Factors	Unit	Minimum		Maximum		Average		Standard Deviation	Tolerance	VIF
Economic Development Level	10,000 yuan/ person		0.293	21.275	4.354		4.458		0.101	9.858
Industrial Structure	%		0.111	0.811	0.437		0.133		0.732	1.383
Urbanization Level	%		0.049	1.000	0.444		0.256		0.413	2.422
Population Size	10,000 people		14.200	152.270	56.635		29.887		0.849	1.178
Population Density	people/hm²		1.540	114.225	14.608		23.379		0.052	19.065
Land Use Structure	%		0.005	0.981	0.159		0.231		0.070	14.215
Consumption Level	10,000 yuan/ person		0.084	18.433	1.757		2.681		0.394	2.538
Investment Level	10,000 yuan/ person		0.028	15.908	2.975		2.972		0.267	3.744

Except for the economic development level, population density, and the land use structure, the tolerance and VIF values of the remaining factors are all the values after excluding the collinear factors

## Results

### Land use change

**Fig. 2:**
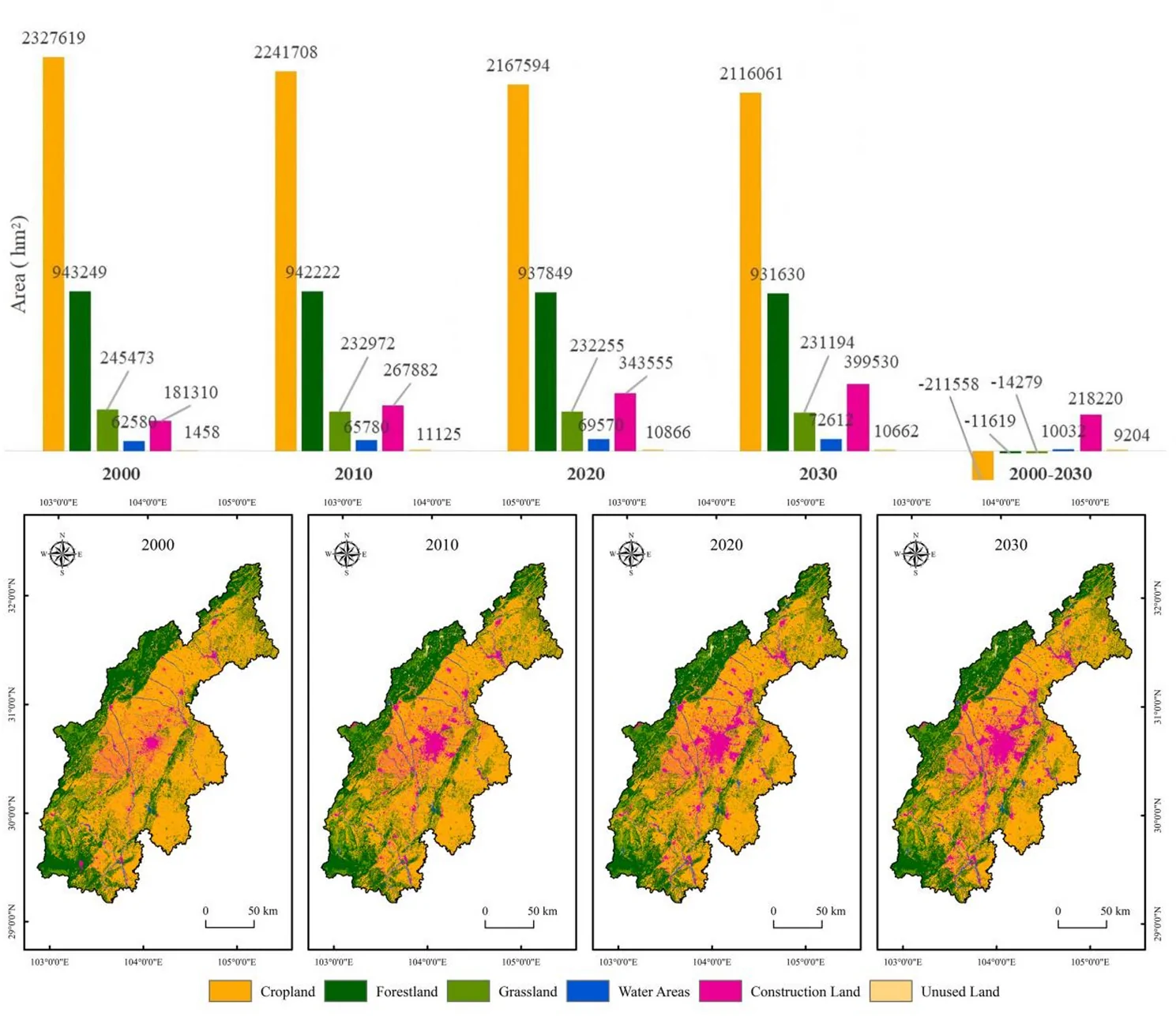
2000–2030 predicted Land Use Change in Chengdu Plain (Statistical chart part: Changes in the area of various land use types in the Chengdu Plain from 2000 to 2030; Map part: Spatial changes of various land use types in the Chengdu Plain from 2000 to 2030.)

In the Chengdu Plain region, cropland and forestland constitute the primary land use types. Despite continuous contraction in their areas in recent years, they still account for over 80% of the total land area, while water areas and unused land occupy relatively small proportions (Fig. 2). As shown in Fig. 2, against the backdrop of rapid urbanization, land use changes in the Chengdu Plain are mainly characterized by the drastic expansion of construction land, the reduction of cropland, forestland, and grassland areas, as well as the corresponding increase in water areas and unused land areas.

Specifically, between 2000 and 2030, the cropland area is projected to decrease from 2,327,619 hm^2^ to 2,116,061 hm^2^, with a total reduction of 211,558 hm^2^ and a change rate of −5.62%. Forestland and grassland areas are expected to decrease by 11,619 hm^2^ and 14,279 hm^2^ respectively, with change rates of −0.31% and − 0.38%. In contrast, the construction land area is expected to grow from 181,310 hm^2^ to 399,530 hm^2^, accumulating an increase of 218,220 hm^2^, ranking first among all land use types in terms of change rate at approximately 5.80%. Water areas and unused land areas will increase by 10,032 hm^2^ and 9,204 hm^2^ respectively.

### Spatial-temporal patterns of land use carbon budgets

To project land use carbon emissions for 2030, this study used SPSS statistical software to conduct time-series linear regression analysis on nearly 20 years of county-level GDP data and a decade of energy consumption data. By analyzing these datasets, researchers developed a predictive model for GDP and energy consumption in 2030. The model was then applied to calculate and forecast the land use carbon emissions of the Chengdu Plain in 2030.

#### Temporal change analysis

**Fig. 3:**
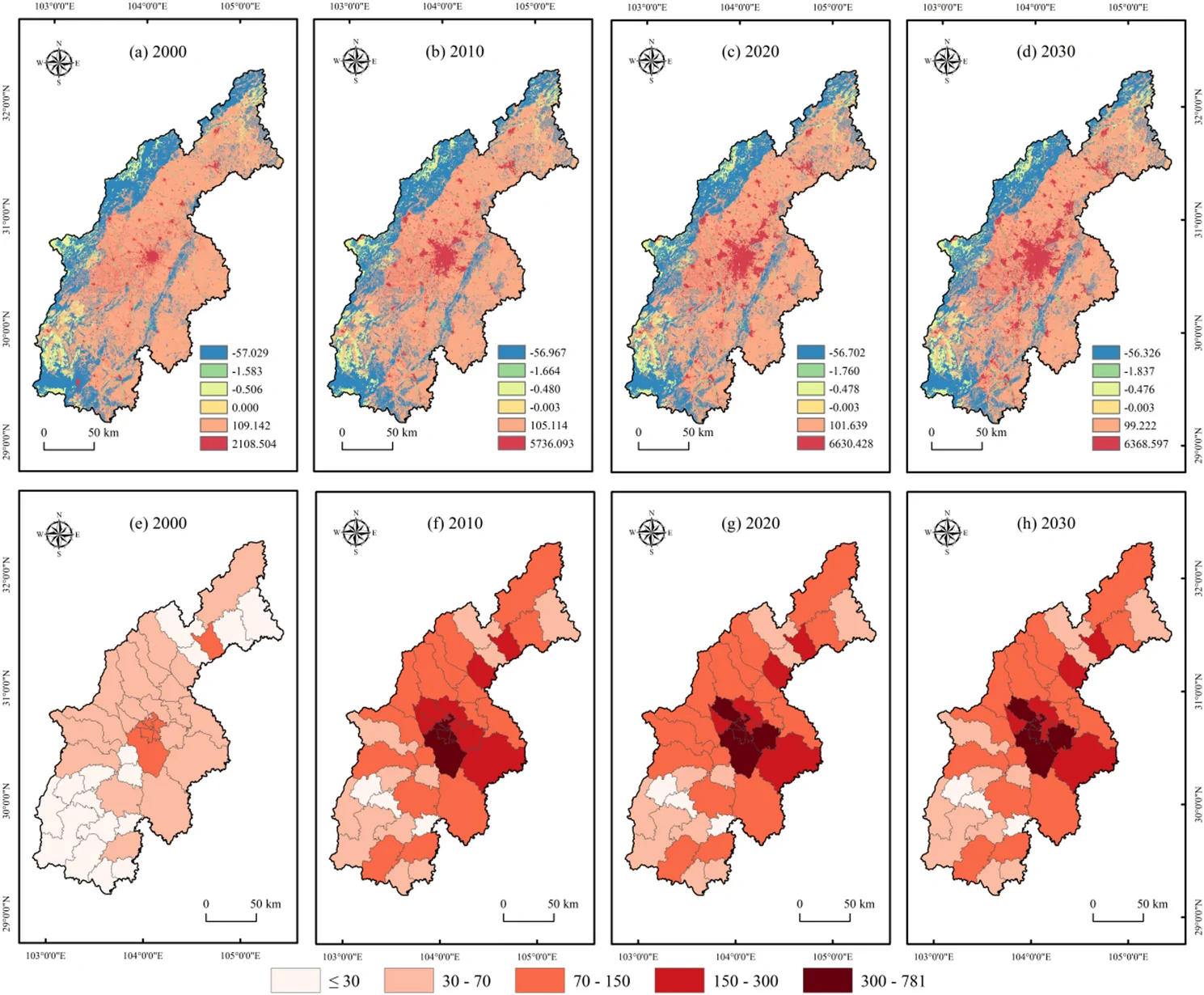
2000–2030 predicted Land Use Carbon Emissions in Chengdu Plain (Unit: 10,000 Tons). (**a-d**) Carbon emissions of different land use types; (**e-f**) Classification of carbon emission levels of land use in each district and county

This study adopted the carbon emission coefficient method and energy accounting approach to quantitatively analyze the carbon emissions (and absorption) from land use in the Chengdu Plain between 2000 and 2030, further exploring their spatiotemporal distribution patterns. The findings, detailed in Table 6; Fig. 3, use positive values to denote carbon emissions and negative values for carbon absorption. Analysis showed that net land use carbon emissions in the region first increased and then decreased: net emissions stood at 2158.528 × 10⁴ t in 2000, rose to 6673.122 × 10⁴ t by 2020, and are projected to drop to 6409.177 × 10⁴ t by 2030. Total carbon emissions followed a similar trajectory—initially rising before declining—while carbon absorption showed relatively minor fluctuations with an overall gradual downward trend.

**Table 6:** Carbon Emissions and Carbon Absorption from Land Use in the Chengdu Plain, 2000–2030 (Unit: 10,000 tons)

Year	Carbon Emissions			Carbon Absorption					Net Carbon Emissions
	Cropland	Construction Land	Total	Forestland	Grassland	Water Areas	Unused Land	Total	
2000	109.142	2108.504	2217.646	−57.029	−0.506	−1.583	0.000	−59.118	2158.528
2010	105.114	5736.093	5841.207	−56.967	−0.480	−1.664	−0.003	−59.114	5782.093
2020	101.639	6630.428	6732.066	−56.702	−0.478	−1.760	−0.003	−58.944	6673.122
2030	99.222	6368.597	6467.819	−56.326	−0.476	−1.837	−0.003	−58.643	6409.177

In terms of the carbon source-sink budget, construction land served as the dominant carbon source, with its emissions increasing from 2108.504 × 10⁴ t in 2000 to 6368.597 × 10⁴ t in 2030, contributing over 98% of total net carbon emissions throughout the study period. Cropland emissions exhibited a slight decline, from 109.142 × 10⁴ t to 99.222 × 10⁴ t, due to area contraction. On the sink side, forestland dominated carbon uptake, accounting for more than 96% of total sequestration capacity, followed by water areas and grassland; unused land contributed negligibly. Despite this, the total carbon sink capacity remained critically insufficient—offsetting less than 1% of gross emissions—highlighting the severe carbon budget imbalance in the region. Notably, with projected technological advancements and industrial restructuring, net carbon emissions are expected to decline after the 2020 peak, signaling a potential decoupling of economic growth from carbon emissions.

#### Spatial change analysis

To facilitate analysis, this study classified land use carbon emissions at the county level into five categories [41]: ① Low-emission zones (annual carbon emissions below 30 × 10^4^ t); ② Moderate Low-emission zones (annual carbon emissions between 30 × 10^4^ t and 70 × 10^4^ t); ③ Moderate-emission zones (annual carbon emissions between 70 × 10^4^ t and 150 × 10^4^ t); ④ High-emission zones (annual carbon emissions between 150 × 10^4^ t and 300 × 10^4^ t); ⑤ Extremely high-emission zones (annual carbon emissions exceeding 300 × 10^4^ t).

From the county scale perspective (Fig. 3), land use carbon emissions in the Chengdu Plain exhibited significant spatial heterogeneity, with an overall distribution pattern of higher emissions in the central region and lower emissions in the north and south. Moderate-emission and higher zones showed a high degree of spatial concentration.

Examining interannual changes, in 2000 most counties across the Chengdu Plain had land use carbon emissions below 700,000 tons (Fig. 3e), with seven moderate-emission zones identified. These included Chengdu’s Jinjiang, Qingyang, Jinniu, Wuhou, Chenghua, and Shuangliu districts, along with Mianyang’s Fucheng District. As urban core areas within the rapidly developing prefecture-level cities of Chengdu and Mianyang, these districts underwent earlier industrialization and more rapid urban expansion than surrounding counties. The intensive development of construction land and infrastructure projects in these areas led to substantial energy consumption, resulting in notably higher carbon emissions during this period compared to neighboring regions. In contrast, low-emission zones were primarily located in the southwest and northeast parts of the plain. These counties generally had less advanced economic development, later industrialization phases, and slower urban growth rates than high-emission areas. Consequently, their energy use remained lower, contributing to correspondingly lower carbon emissions from land use activities.

From 2000 to 2010, the spatial distribution of land use carbon emissions across the Chengdu Plain saw notable transformations (Fig. 3e and f). Except for Danling County, Qingshen County, and Mingshan District, 40 of the 43 counties experienced significant emission increases. Among these, 29 counties reached moderate or higher emission tiers, with five districts—Chengdu’s Jinjiang, Qingyang, Jinniu, Wuhou, and Shuangliu—emerging as extremely high-emission zones. Counties with the most pronounced emission changes clustered primarily in Chengdu’s municipal districts and other prefecture-level city centers. Fueled by sustained economic growth and industrial expansion, accelerated urbanization, rapid construction land expansion, and surging energy demand drove drastic rises in land use carbon emissions across these regions.

Between 2010 and 2020, most counties in the Chengdu Plain maintained stable carbon emission categorizations, with only a few exceptions: Dayi County transitioned from a lower-emission tier to moderate emissions, while Longquanyi and Pidu districts entered the extremely high-emission category. During this decade, 17 counties recorded declining land use carbon emissions, and 22 saw slow growth—only Wuhou, Longquanyi, Shuangliu, and Pidu districts experienced notable rises. A deeper analysis of the emission decreases in certain counties highlights recent technological advancements and expanded adoption of new energy sources as key drivers. Conversely, the growth in the four districts with increasing emissions is tied to distinct regional dynamics: Wuhou, Shuangliu, and Pidu districts benefited from rapid development in Chengdu’s High-tech Industrial Zone, while Longquanyi District’s surge stemmed from industrial clustering in automobile manufacturing, which amplified energy demands and associated emissions.

According to the 2030 projection results, compared to 2000, carbon emissions in all 43 counties are expected to increase, ranging from 13.263 × 10^4^ t to 556.507 × 10^4^ t. Wuhou District is projected to have the largest emission increase, while Danling County the smallest. Although the regional classification of emission categories is expected to remain largely stable by 2030, most counties are forecast to see lower carbon emissions than in 2020, with limited growth rates in areas where emissions continue rising. The anticipated reduction in the Chengdu Plain’s carbon emissions can be attributed to key factors: widespread new energy adoption, optimization of industrial structures, and strengthened ecological protection measures. These initiatives are playing a crucial role in curbing emissions across the region.

### Spatial autocorrelation analysis of land use carbon budgets

#### Global spatial autocorrelation analysis

Considering the attenuation effect of geographical distance on regional associations and adapting to the characteristic of the Chengdu Plain that “the closer the distance, the stronger the spatial interaction”, when using the ArcGIS spatial autocorrelation tool to calculate the global Moran’s *I* index, we selected the INVERSE_DISTANCE concept to conceptualize the spatial relationship and calculated the distance between county centroids using the Euclidean distance to construct the weights. According to the analysis results of the global Moran’s *I* index of land use carbon emissions in the Chengdu Plain from 2000 to 2030 (Table 7), the Moran’s *I* values for the four investigated years are all positive (greater than 0) and have all passed the 99% significance level test. This result reveals that there is a significant positive spatial correlation among the land use carbon emissions of each county (district) in this region. That is, counties (districts) with high or low carbon emissions tend to cluster adjacent to each other in geographical space.

**Table 7:** Global Moran’s *I* Index of Land Use Carbon Emissions in the Chengdu Plain

Year	2000	2010	2020	2030
Moran’s *I*	0.427	0.590	0.488	0.534
Z-score	4.696	6.336	5.856	6.057
P-value	0.000	0.000	0.000	0.000

#### Local spatial autocorrelation analysis

**Fig. 4:**
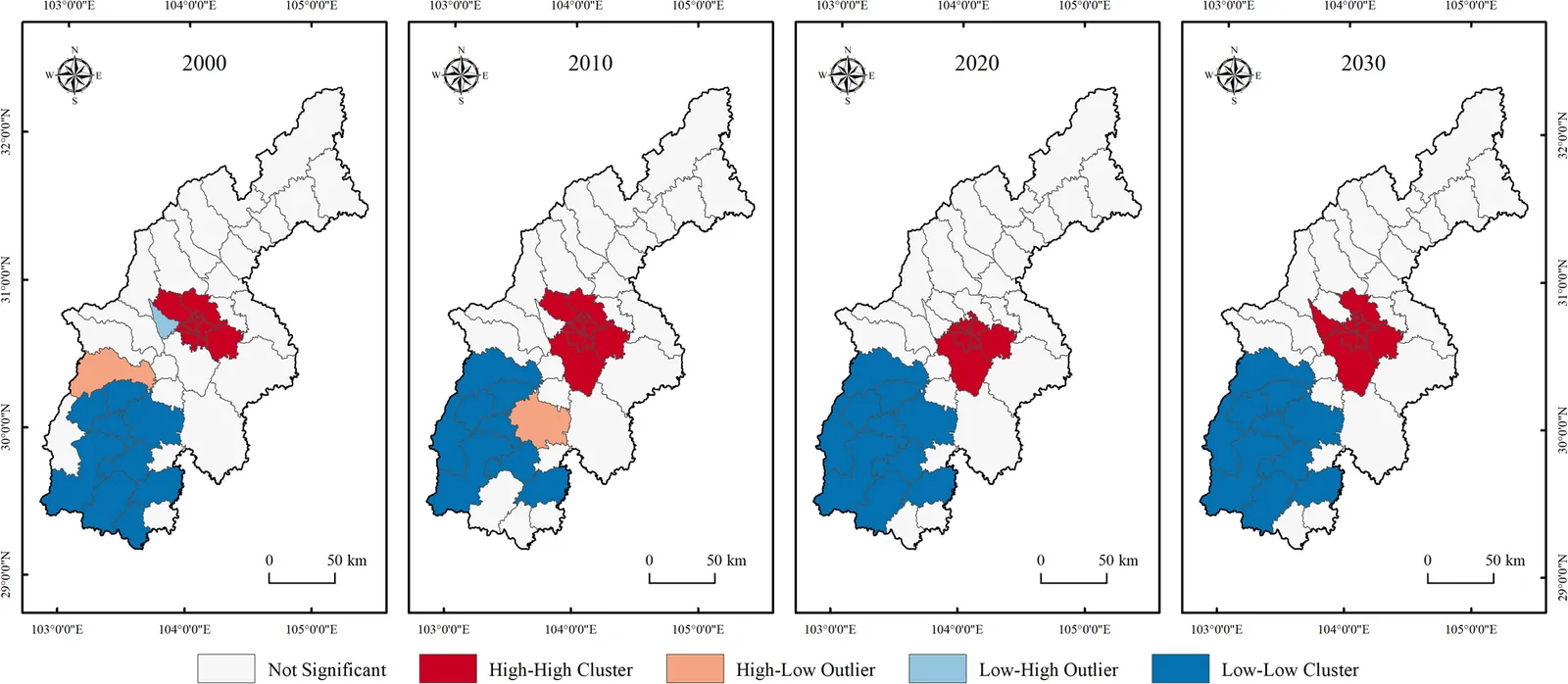
Evolution of Local Spatial Agglomeration Patterns of Land Use Carbon Emissions in the Chengdu Plain

During the period from 2000 to 2030, the results of the local autocorrelation analysis of land use carbon emissions in the Chengdu Plain region are shown in Fig. 4. Overall, the spatial agglomeration patterns of land use carbon emissions in the counties and districts of this region were mainly characterized by High-High (HH) clustering and Low-Low (LL) clustering, and their spatial distribution patterns remained relatively stable. Specifically, the Chengdu municipal districts, which are relatively economically developed, formed the core area of high-value agglomeration, while the southwestern counties with less developed economies formed a low-value agglomeration zone. During the study period from 2000 to 2030, the number of High-High (HH) clustering areas changed little, but showed a trend of expanding to peripheral areas. For example, Wenjiang District was still in the low-value center in 2000, but it is expected to transform into a High-High (HH) clustering area by 2030. This change is closely related to the expansion of urbanization in Chengdu and the transfer of industries to surrounding peripheral counties. Industrial transfer has promoted the transformation of Wenjiang District from a traditional agricultural and light manufacturing industry-dominated industrial structure to a diversified industrial pattern including biopharmaceuticals, equipment manufacturing, and modern service industries. At the same time, the distribution of Low-Low (LL) clustering areas was also relatively stable, mainly concentrated in the southwestern counties. These counties have relatively slow economic development, an industrial structure dominated by agriculture, and low carbon emissions, thus becoming low-value agglomeration areas.

### Analysis of influencing factors on land use carbon budgets

#### Comparison of multi-model fitting results

Using the Geographically and Temporally Weighted Regression (GTWR) module in ArcGIS 10.8 software, this study calculated the regression coefficients and model fitting parameters for influencing factors under different models (Table 8). The analysis results showed that the adjusted R-squared of the GTWR model was 0.945 (greater than 0.9), which not only exceeded that of the GWR model but also was higher than that of the Ordinary Least Squares (OLS) model. In addition, both the Residual Sum of Squares (RSS) and the Akaike Information Criterion (AIC) of the GTWR model were lower than those of the OLS and GWR models. In summary, the GTWR model significantly outperformed the OLS and GWR models in terms of fitting effectiveness, allowing for a more accurate assessment of the impact of various influencing factors on land use carbon emissions in the Chengdu Plain.

**Table 8:** Comparison of Fitting Performance Among Different Models

Model	*R* ^2^	Adjusted *R*^2^	AIC	RSS
OLS	0.801		167.612	25.431
GWR	0.914	0.911	172.332	11.073
GTWR	**0.945**	**0.942**	**162.010**	**7.140**

Bold values indicate the best-performing model for each evaluation criterion. Adjusted R², AIC (Akaike Information Criterion), and RSS (Residual Sum of Squares) were used as goodness-of-fit measures

#### Analysis of GTWR regression results

**Fig. 5:**
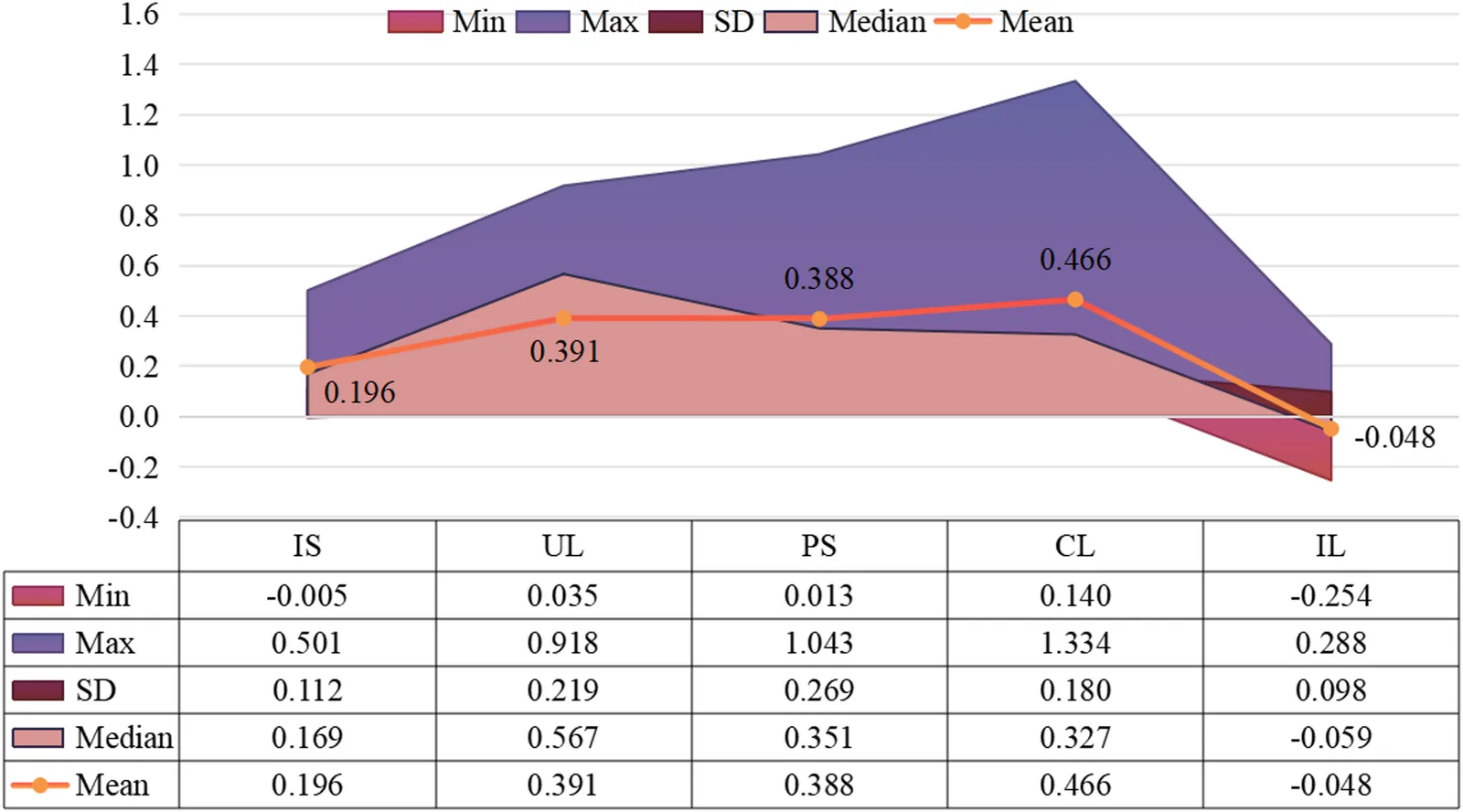
Descriptive Statistics of Regression Coefficients (The upper part of the diagram shows the size distribution of each statistical indicator, and the table below indicates its specific values. Min refers to the minimum value, Max refers to the maximum value, SD refers to the standard deviation, Median refers to the median, and Mean refers to the mean value.)

This study employed the GTWR analysis module in ArcGIS software to conduct a spatio - temporal geographically weighted regression analysis on each district and county in the Chengdu Plain to calculate the regression coefficients of various influencing factors under different time series. Specifically, the model was constructed based on panel data of 43 districts and counties × 3 time nodes (the years 2000, 2010, and 2020), forming an observation matrix containing 129 spatiotemporal sample points to support the analysis of the spatiotemporal heterogeneity of regression coefficients. Subsequently, the obtained regression results were imported into SPSS software for processing. By selecting statistical indicators such as minimum, maximum, standard deviation, mean, and median, a detailed descriptive statistical analysis of the regression coefficients was conducted. The analysis results revealed the descriptive statistical characteristics of the regression coefficients of the GTWR model in the Chengdu Plain (Fig. 5). According to the mean analysis results, the influencing factors generally had a positive promoting effect on land use carbon emissions in the Chengdu Plain (with the exception of investment level). Among them, the regression coefficient of consumption level was the highest (0.466), and the order of regression coefficients was as follows: consumption level (CL) > urbanization level (UL) > population size (PS) > industrial structure (IS) > investment level (IL). However, based on the median analysis results, urbanization level had the most significant impact on land use carbon emissions in the Chengdu Plain, with a regression coefficient of 0.567. The order of regression coefficients was as follows: urbanization level (UL) > population size (PS) > consumption level (CL) > industrial structure (IS) > investment level (IL). Comprehensive analysis indicated that urbanization level, consumption level, and population size had relatively significant impacts on land use carbon emissions in the Chengdu Plain, while the impacts of industrial structure and investment level were relatively small.

#### Spatiotemporal heterogeneity of influencing factors

**Fig. 6:**
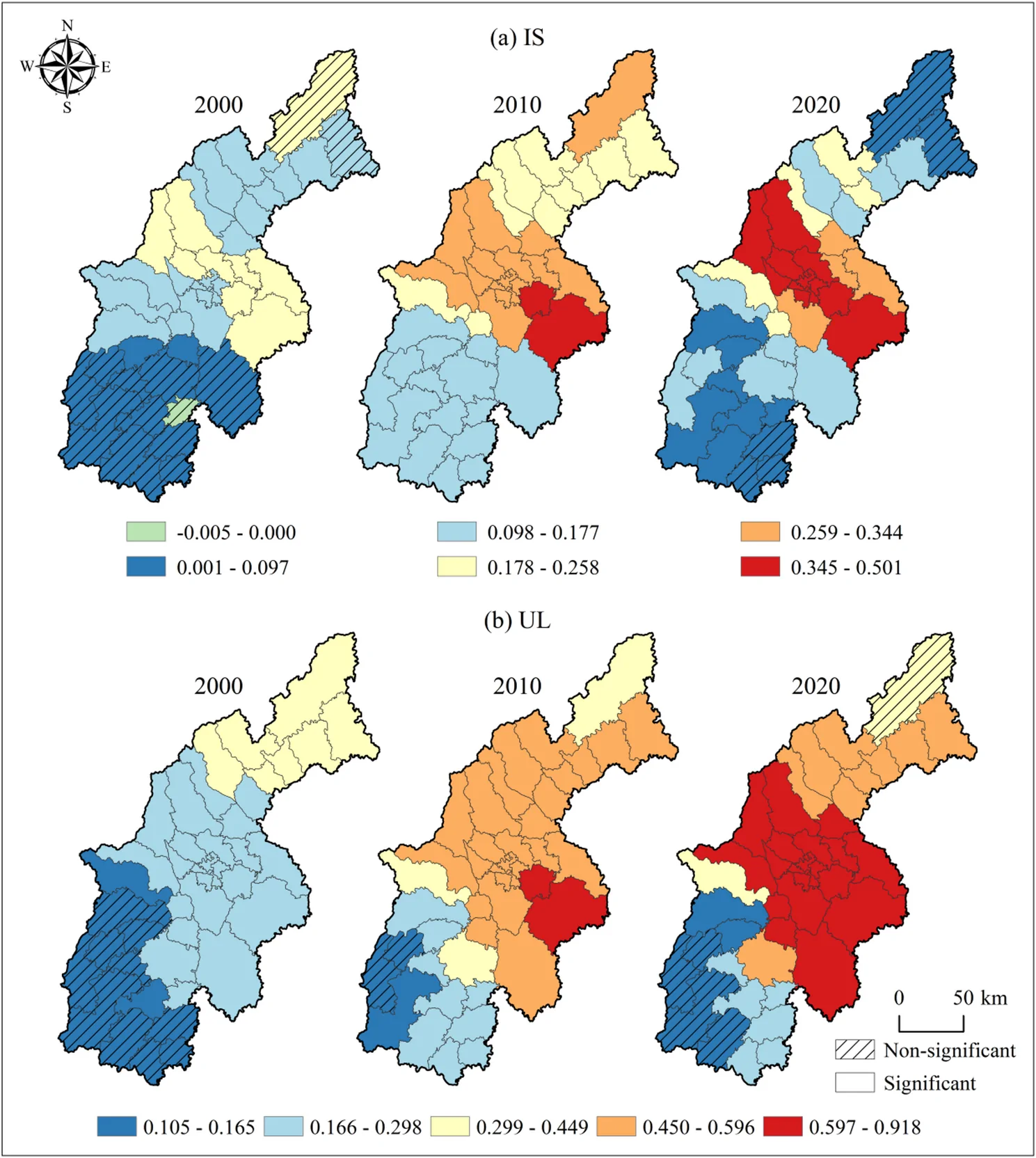
Spatiotemporal Distribution of Regression Coefficients for Industrial Structure (IS) and Urbanization Level (UL)

**Fig. 7:**
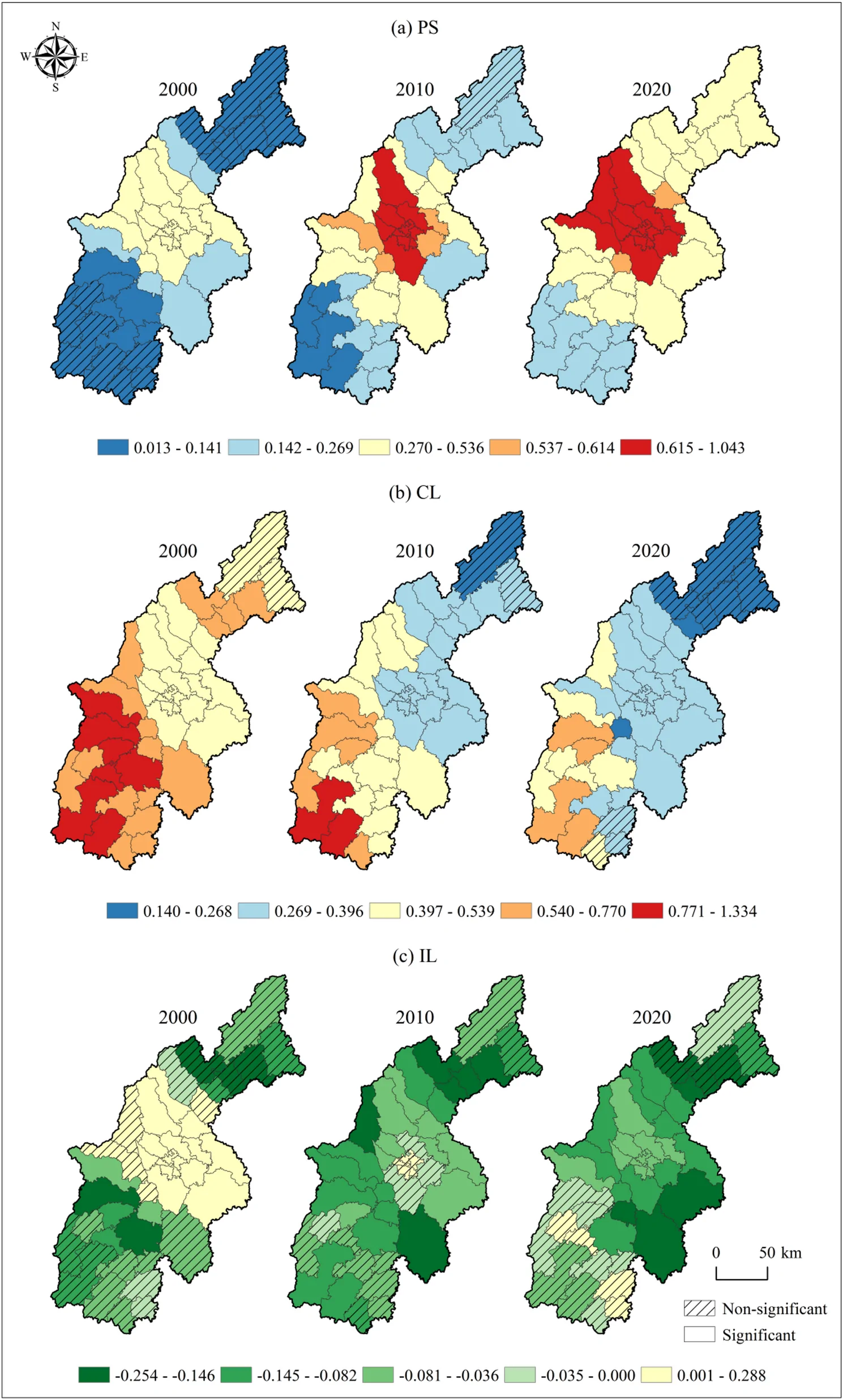
Spatiotemporal Distribution of Regression Coefficients for Population Size (PS), Consumption Level (CL), and Investment Level (IL)

The regression coefficients of each influencing factor were spatially visualized using ArcGIS, classified into 5–6 levels via the natural breaks method (Figs. 6 and 7). Positive coefficients indicate a promoting effect on net carbon emissions, while negative coefficients indicate an inhibiting effect. All coefficients discussed below passed the significance test at the 0.05 level unless otherwise noted.

1. Industrial Structure (IS)

Industrial structure refers to the proportion of the output value of the secondary industry in the total regional output value. There are significant differences in energy consumption density and structure among different industries, leading to regional differentiation in carbon emissions caused by differences in industrial structure [49]. It exerted an overall positive effect on net carbon emissions (coefficient range: −0.005 to 0.501), displaying a spatial pattern of higher values in central counties and lower values in northern and southern peripheries. The high-value areas were concentrated in Chengdu’s urban districts and adjacent counties such as Jianyang, Dujiangyan, and Pengzhou—regions that have absorbed industrial transfer from Chengdu’s core. Southern counties under Ya’an, Leshan, and Meishan showed the weakest influence, reflecting their slower secondary industry development. Temporally, coefficients in central counties exhibited a gradual upward trend, driven by intensifying production activities and associated coal, water, and land resource consumption.

(2) Urbanization Level (UL)

Urbanization level refers to a quantitative indicator of the degree of urbanization in a region. Generally, as the urbanization rate increases, the expansion of construction land area alters the economic development model and efficiency of cities and towns, which in turn leads to an increase in carbon emissions [22, 50, 51]. It showed a strong positive effect on net carbon emissions (coefficient range: 0.105–0.918), with a spatial pattern of higher values in central-northern counties and lower values in the southwest. Counties most affected included those in central Chengdu, Deyang, and Mianyang; southern counties such as Emeishan, Jiajiang, and Qingshen were relatively less impacted. This spatial differentiation aligns with observed and projected construction land expansion: from 2000 to 2020, northern counties with high UL coefficients experienced significantly greater built-up land growth than southern counties with low UL coefficients, and FLUS projections for 2030 indicate that this differential trend will persist. Temporally, coefficients in most central-northern counties increased year by year, while those in southern regions remained stable or declined—a pattern consistent with the concentration of three major cities (Chengdu, Deyang, Mianyang) in the north, where urbanization has proceeded considerably faster.

(3) Population Size (PS)

Population size positively affected net carbon emissions across all counties (coefficient range: 0.013–1.043), with coefficients decreasing from the center to the periphery. The highest coefficients were concentrated in Chengdu’s core urban districts (e.g., Jinjiang, Chenghua, Wuhou, Wenjiang), while lower values were observed in Hongya and Emei in the south, and Mianzhu and Anzhou in the north. Temporally, coefficients in northern counties (Zitong, Luojiang, Fucheng) and central Chengdu showed an overall increasing trend, reflecting intensified human activity impacts, whereas southern counties remained largely stable. This spatial pattern is consistent with the trajectory of construction land expansion: population agglomeration in core districts drove rapid built-up growth from 2000 to 2020 [52], and the FLUS-projected slowdown in expansion by 2030 corresponds to the stabilizing PS coefficients observed in GTWR, suggesting the population-driven emission effect may plateau as urban expansion decelerates.

(4) Consumption Level (CL)

In this study, the Consumption Level (CL) is represented by the total retail sales of consumer goods per capita. As the standard of living improves, the demand for consumer goods continues to grow, which in turn promotes the production of consumer products and leads to an increase in carbon emissions [53]. CL exhibited the highest mean regression coefficient among all factors (0.466, range: 0.140–1.334), indicating its dominant role in driving net carbon emissions. In contrast to urbanization and population, its spatial pattern was reversed: coefficients were substantially higher in southern and western counties (e.g., Hongya, Emei, Pujiang, Qionglai, Dayi) than in central-eastern and northern regions (e.g., Anzhou, Luojiang, Fucheng, Youxian). This reflects the fact that in less-economically-developed counties, large-scale consumption of consumer goods contributes disproportionately to local carbon emissions relative to other factors. Temporally, CL coefficients declined markedly across all counties from 2000 to 2020, suggesting that the marginal carbon impact of consumption is diminishing with economic development.

(5) Investment Level (IL)

Investment level was unique among the five factors in exhibiting an overall negative mean coefficient (−0.048), indicating an inhibiting effect on net carbon emissions in most counties, particularly in central and eastern regions. However, the regression fit was less ideal compared to other factors. Spatially, coefficients were higher (less negative or slightly positive) in Chengdu’s core districts and lower (more negative) in peripheral counties. Temporally, coefficients shifted from positive to negative and continued to decline throughout the study period. This temporal evolution likely reflects a structural shift in investment composition: early investments were concentrated in high-emission secondary industries, while recent years have seen increasing proportions directed toward low-carbon sectors, environmental governance, and clean energy—consistent with the observation that counties with high “industrial technological transformation” investment (e.g., Jianyang, Renshou) show the strongest IL inhibiting effect.

In summary, the GTWR analysis reveals a clear spatial division of driving mechanisms: urbanization and population effects dominate central-northern counties aligned with the Chengdu-Deyang-Mianyang urban corridor, while consumption effects dominate the less-urbanized southwestern counties. This heterogeneity provides the empirical foundation for the county-specific carbon management strategies discussed in Sect. "Differentiated carbon balance management strategies based on drivingmechanism heterogeneity".

## Discussion

This study investigated the spatiotemporal patterns and driving mechanisms of county-level land use carbon budgets in the Chengdu Plain by integrating FLUS simulation, spatial autocorrelation analysis, and GTWR modeling. The results reveal three key findings with direct implications for carbon balance management: a projected carbon peak around 2020, spatially heterogeneous driver effects that challenge uniform policy approaches, and stable spatial agglomeration patterns that justify cross-county coordination. These findings collectively provide an empirical basis for refining county-scale carbon management in rapidly urbanizing yet ecologically critical regions.

### Temporal and spatial distribution characteristics of net carbon emissions from land use in the Chengdu plain

Against the backdrop of rapid urbanization and a sharp expansion in the scale of construction land, there have been significant changes in land use in the Chengdu Plain. Its net carbon emissions are characterized by higher levels in the central part and lower levels in the north and south, showing a trend of first increasing and then decreasing, which is consistent with the research findings of Peng et al. [8]. The reason for this characteristic is that the industrialization in the central districts and counties started earlier, and the urbanization process was more rapid. The development and construction of construction land consumed a large amount of energy, resulting in higher net carbon emissions during the same period compared to other surrounding districts and counties. The trend of net carbon emissions first increasing and then decreasing is closely related to the regional economic transformation and industrial structure optimization. With the proposal of the “dual carbon” goal, the Chengdu Plain has actively responded by vigorously promoting the adjustment of the industrial structure, gradually reducing the proportion of high energy consuming industries, and increasing the proportion of clean energy use. For example, in recent years, the Chengdu Plain has increased support for low energy consuming and high value added industries such as electronic information and biomedicine, reducing its dependence on traditional high energy consuming industries and effectively controlling the growth rate of net carbon emissions. At the same time, the implementation of regional ecological policies, such as strengthening forest protection and afforestation activities, has enhanced the carbon sequestration capacity of forest land, further contributing to the reduction of net carbon emissions.

### Spatial autocorrelation of land use net carbon emissions in the Chengdu Plain

The net carbon emissions of land use in the Chengdu Plain exhibits spatial characteristics of High-High clustering and Low-Low clustering. The high-value clustering areas are mainly concentrated in the economically developed urban districts of Chengdu. In these areas, the levels of urbanization and industrialization are high, the scale of construction land is large, and the energy consumption is significant, resulting in large net carbon emissions that is clustered in close proximity to each other. For example, in the urban districts of Chengdu, industrial activities such as high-tech industries and manufacturing are intensive, with a strong demand for energy, keeping net carbon emissions at a high level. On the other hand, the low-value clustering areas are concentrated in the economically underdeveloped districts and counties in the southwest. These areas are dominated by agriculture, with relatively backward industrial development, low energy consumption, and thus low net carbon emissions.

### County-level heterogeneity of influencing factors of land use carbon budgets in the Chengdu Plain

The GTWR results indicate that the magnitude and direction of influencing factors vary substantially across counties and over time. Compared with traditional macro-scale studies, the county-scale analysis reveals the following characteristics that have been largely overlooked:

1. Cross-scale comparison of dominant driving factors

At the provincial scale, population size, population density, and land use structure have been identified as the dominant factors affecting carbon emissions, with mean regression coefficients of 0.554, 0.610, and 0.720, respectively [54]. At the municipal scale, population size, the gross output value of the primary and tertiary industries, and the irrigated area of cultivated land are the dominant factors, with regression coefficients of 0.823, 0.817, 0.777, and 0.839, respectively [21]. At the county scale investigated in this study, the dominant factors are consumption level (0.466), urbanization level (0.392), and population size (0.388). Population size consistently emerges as a dominant factor across all three scales, underscoring its fundamental role in driving regional carbon emissions. The divergence in other dominant factors between scales highlights the scale-dependence of carbon emission driving mechanisms and confirms the unique value of county-level analysis in capturing drivers that macro-scale studies inevitably average out. This finer resolution provides a critical basis for designing spatially precise carbon management measures.

(2) Spatial differentiation of influencing factors and its implications

Although the mean regression coefficient identifies consumption level (0.466) as the primary driver of net carbon emissions across the Chengdu Plain, county-level disaggregation reveals a far more nuanced picture. The consumption coefficients of southwestern counties are mostly above 0.540, with some exceeding 1 (e.g., Qionglai: 1.334; Pujiang: 1.017), whereas those of central-northern urban core counties are predominantly below 0.396. This spatial inversion reflects the fact that in less-economically-developed counties, the scale of consumer goods consumption contributes disproportionately to local carbon emissions relative to other factors.

Urbanization level presents a contrasting spatial pattern. Its coefficients in central-northern urban core counties are generally high, with most exceeding 0.449, while southwestern counties exhibit substantially lower coefficients, with some falling below 0.165 (e.g., Hongya, Jiajiang). This indicates that urban expansion and infrastructure construction in the central-northern region exert a considerably stronger marginal effect on net carbon emissions, driven by more intensive energy demand and concentrated industrial activities. In the southwestern region, where urbanization lags behind, its marginal contribution to carbon emissions remains weak.

Population size also displays marked spatial differentiation. Coefficients in central and northern counties are generally high (mostly around 0.536), suggesting that population growth exerts a more prominent promoting effect on net carbon emissions in these areas—likely linked to rapid economic development, high population density, and frequent consumption activities. In contrast, southwestern counties show lower coefficients, which may be attributable to population outflow, accelerating population aging, and lagging economic growth.

In addition, by integrating the driving heterogeneity identified by GTWR with the land use trajectories projected by FLUS for 2030, the following preliminary predictions can be derived: ① In urban-core counties (e.g., Jinjiang, Jinniu), the projected slowdown in construction land expansion may alleviate the driving heterogeneity of population size (PS) and urbanization level (UL), potentially narrowing the coefficient gap between core and peripheral counties. ② In industrial counties (e.g., Longquanyi, Jianyang), continued construction land expansion may intensify the driving heterogeneity of industrial structure (IS), likely expanding the spatial extent of high IS coefficient areas. ③ In less-developed southwestern counties (e.g., Hongya, Jiajiang), where construction land expansion is projected to remain stable, the driving heterogeneity of consumption level (CL) is expected to persist.

Collectively, these differences demonstrate that macro-scale “averaged” coefficients cannot capture the functional diversity of county-level units. A fine-grained county-scale analysis is essential to accurately identify the core drivers of net carbon emissions in different county types, thereby providing the micro-level evidence base required for differentiated carbon balance management strategies.

### Differentiated carbon balance management strategies based on driving mechanism heterogeneity

The GTWR results reveal that the driving mechanisms of net carbon emissions differ fundamentally across county types. Uniform mitigation targets are therefore unlikely to be effective. Based on the dominant influencing factors identified for each county category, we propose the following spatially differentiated carbon balance management strategies.

1. Urban-core counties (central Chengdu districts): Coordinated “population–urbanization” carbon management

Population size and urbanization level are the primary drivers of net carbon emissions in these counties, with most regression coefficients exceeding 0.596 since 2020. Specifically, PS coefficients in Jinjiang, Jinniu, and Wuhou all surpassed 0.950 in 2020 and continue to rise, reflecting the significant carbon pressure from population agglomeration. The UL coefficient in Jianyang reached 0.918, and FLUS simulations project continued construction land expansion with ongoing urbanization, indicating that net carbon emissions pressure will intensify. For these counties, carbon balance management should prioritize: (i) compact urban form optimization through mixed-use zoning of commercial, residential, and public service land to reduce commuting-related carbon emissions; and (ii) low-carbon community retrofitting in high-density areas, including building-integrated solar panels and community-level smart energy management systems to reduce per-capita residential energy consumption.

(2) Industrial-dominant counties (e.g., Longquanyi, Jianyang, Pengzhou): Industrial structure adjustment with carbon intensity regulation

These counties exhibit relatively high IS coefficients (Jianyang: 0.501; Longquanyi: 0.454), indicating a significant dependence on high-energy-consumption industries. The rapid growth of the secondary sector has made industrial land the dominant emission source. For these counties, carbon management should focus on: (i) carbon intensity thresholds for industrial land—setting maximum allowable carbon emissions per unit area of industrial land and phasing out enterprises exceeding the standard; (ii) green industrial park development that encourages upstream-downstream enterprise agglomeration to reduce transportation emissions; and (iii) tilting industrial land quotas toward low-carbon sectors such as biomedicine and clean energy equipment manufacturing, thereby optimizing the structure of industrial land use

(3) Agro-ecological counties in the southwest (e.g., Hongya, Jiajiang): Green consumption promotion and sink enhancement

In these counties, the CL regression coefficients are significantly higher than those of other factors. Although CL coefficients declined from 2000 to 2020, consumption level remains the dominant driver of net carbon emissions. Additionally, PS coefficients show an upward trend, indicating the growing impact of population size. Carbon balance management in these counties should emphasize: (i) green consumption incentives, encouraging residents to choose low-carbon products and services through subsidies or policy incentives; (ii) rational population distribution planning to avoid emission increases from excessive population concentration; and (iii) forest and cropland sink protection through ecological red-line policies, given these counties’ important role in the regional carbon sink network.

(4) Cross-county carbon compensation and green investment mechanisms

The GTWR results show that investment level exerts an inhibiting effect on net carbon emissions (mean coefficient: −0.048), particularly in counties with a high proportion of environmental governance and clean energy investment (e.g., Jianyang, Renshou). This finding provides an empirical basis for designing carbon management instruments at the regional scale: (i) green finance guidance—directing funds toward environmental governance and clean energy projects, with per-capita green investment benchmarks set for each county type; (ii) cross-county carbon compensation mechanisms that transfer resources from high-emission urban-core counties to sink-providing agro-ecological counties, using the county-level carbon budget data (Fig. 3) as a quantitative basis for payment calculations; and (iii) targeted low-carbon subsidies for southwestern agricultural counties (e.g., rural photovoltaics, small-scale water conservancy projects) to strengthen the investment inhibiting effect where it is currently weakest.

### Implications for carbon balance management

Beyond the county-specific policy suggestions outlined above, this study offers broader implications for regional carbon balance management, which lies at the core of this journal’s scope.

1. Establishing a county-level carbon budget monitoring framework. Our integration of FLUS simulation with county-scale carbon accounting provides a replicable methodology for constructing carbon budget inventories at sub-municipal levels. Such spatially explicit source-sink accounts can serve as the scientific basis for Monitoring, Reporting, and Verification (MRV) systems required by carbon neutrality policies. By identifying which counties are net carbon sources versus net sinks (Fig. 3), policymakers can establish inter-county carbon compensation mechanisms with transparent, data-driven benchmarks.
2. Addressing heterogeneity in carbon neutrality pathways. The GTWR results (Figs. 6 and 7) demonstrate that uniform, top-down carbon reduction targets may be inefficient or even counterproductive. For instance, applying the same urbanization control policies to central-northern counties (where urbanization strongly drives emissions) and southwestern counties (where consumption level dominates) would fail to address the actual local drivers. Our county typology—urban-core, industrial-dominant, and agro-ecological—provides a practical template for tailoring carbon management instruments to local emission-sink profiles.
3. Redirecting investment as a carbon management tool. The finding that investment level exerts an inhibiting effect on net carbon emissions (mean coefficient: −0.048) in most counties—especially those with high proportions of environmental governance and clean energy projects—offers a powerful policy lever. Green finance and fiscal instruments can be strategically directed to amplify this effect. Specifically, our results support setting per-capita green investment benchmarks and using carbon intensity thresholds for land-use approval, which directly translates carbon balance science into actionable economic governance.

These implications collectively position county-scale carbon budget analysis as a manageable unit for achieving national “dual carbon” goals, especially in regions where economic development and ecological protection must be balanced, such as the upper Yangtze River basin and beyond.

### Limitations and future research directions

Several limitations of this study should be acknowledged. Regarding data, county-level energy consumption was estimated using the unit GDP energy consumption coefficient method (a top-down allocation approach). This method relies solely on GDP shares to apportion energy use across counties, without accounting for industry-specific energy efficiencies or regional peculiarities in consumption patterns. Since energy intensity can vary substantially across high-energy-consumption sectors, this approach may introduce systematic biases into the net carbon emissions estimates. Methodologically, time-series linear regression was employed to project GDP and energy consumption for 2030. While this captures historical temporal trends, it does not incorporate real-world variables and uncertainties—such as public emergencies, macroeconomic fluctuations, or major policy shifts—that may significantly alter future trajectories. Furthermore, the static allocation of energy consumption to construction land is not dynamically coupled with the FLUS-simulated spatial expansion of built-up areas, resulting in a disconnect between the carbon accounting of construction land and the spatially explicit land use projections.

To address these limitations, future research could pursue several directions. First, incorporating diverse data sources—such as sector-disaggregated energy consumption statistics from macroeconomic and industrial databases—would enable a more comprehensive and accurate county-level carbon budget inventory, better capturing sector-specific nuances in energy use. Second, predictive modeling could be strengthened by complementing time-series regression with machine learning approaches (e.g., Random Forest, Support Vector Machine) and multi-model ensemble techniques, which are better suited to handling complex, non-linear relationships and real-world uncertainties. Third, conducting policy scenario analyses—simulating alternative industrial restructuring pathways or energy policy frameworks—would allow for more nuanced and policy-relevant projections of carbon budgets. Finally, methodologically, tighter integration of the FLUS spatial simulation outputs with the carbon accounting framework—for example, by linking construction land expansion trajectories directly to projected energy consumption patterns—would enhance the internal consistency of the modeling framework.

## Conclusion

This study integrated the FLUS model, spatial autocorrelation analysis, and the GTWR model to investigate the county-level carbon budget dynamics and heterogeneous driving mechanisms in the Chengdu Plain from 2000 to 2030. Three key contributions emerge:

1. County-scale carbon budget trajectory with future projection. Net carbon emissions initially increased from 2158.528 × 10^4^ t in 2000 to a peak of 6673.122 × 10^4^ t in 2020, then is projected to decline to 6409.177 × 10^4^ t by 2030. This turning point, revealed through county-level coupling of FLUS-simulated land use and energy-based emission accounting, indicates a potential decoupling of urbanization from net carbon emissions—a signal masked in provincial-scale studies. Construction land dominated the emission source (> 98%), while forests provided the primary sink capacity (> 96%), yet the sink offset remained critically low, underscoring the severe carbon budget imbalance.
2. Spatiotemporally heterogeneous driving mechanisms. GTWR analysis revealed that the effects of key drivers vary significantly across counties and time. Consumption level (0.466), urbanization level (0.392), and population size (0.388) were the dominant positive drivers, displaying clear spatial directionality—urbanization strongly impacted central-northern counties, while consumption level dominated southwestern regions. Critically, investment level (−0.048) exhibited an overall inhibiting effect, particularly in counties receiving substantial green investment, refining the conventional understanding that investment uniformly promotes emissions.
3. County typology-based carbon management framework. Based on the identified heterogeneity, we propose a differentiated management strategy: urban-core counties (e.g., Jinjiang) should prioritize low-carbon urban form optimization; industrial counties (e.g., Longquanyi) require carbon intensity thresholds for industrial land; agro-ecological counties (e.g., Hongya) benefit from green consumption incentives; and cross-county carbon compensation mechanisms should be established, underpinned by red-line policies protecting cropland and forests. This framework translates scientific findings into spatially precise carbon balance governance, offering a transferable paradigm for regions balancing rapid urbanization with ecological security.

## Data Availability

No datasets were generated or analysed during the current study.

## Abbreviations

FLUS: Future land use simulation
GTWR: Geographically and temporally weighted regression
GWR: Geographically weighted regression
LMDI: Logarithmic mean divisia index
IS: Industrial structure
UL: Urbanization level
PS: Population size
CL: Consumption level
IL: Investment level
